# Three-dimensional models of physeal fractures in the femur for the teaching of veterinary medicine

**DOI:** 10.1590/acb395424

**Published:** 2024-08-05

**Authors:** Kleber dos Anjos Lucas, Siham Kassab, Rodrigo Gomes de Souza, Nongnuch Inpanbutr, Marco Aurélio Pereira-Sampaio, Yuri Karaccas Carvalho

**Affiliations:** 1 Universidade Federal do Acre – Centro de Ciências Biológicas e da Natureza – Rio Branco (AC) – Brazil.; 2 The Ohio State University – Department of Veterinary Clinical Sciences – Columbus (OH) – United States of America.; 3 Universidade do Estado do Rio de Janeiro – Urogenital Research Unit – Rio de Janeiro (RJ) – Brazil.; 4 Universidade Federal Fluminense – Department of Morphology – Niterói (RJ) – Brazil.; 5 Universidade Federal Fluminense – Department of Pathology and Veterinary Clinic – Niterói (RJ) – Brazil.

**Keywords:** Printing, Three-Dimensional, Anatomy, Orthopedics, Salter-Harris Fractures, Teaching Materials

## Abstract

**Purpose:**

To develop and assess three-dimensional models of physeal fractures in dog femurs (3D MPFDF) using radiographic imaging.

**Methods:**

The study was conducted in three phases: development of 3D MPFDF; radiographic examination of the 3D MPFDF; and comparative analysis of the anatomical and radiographic features of the 3D MPFDF.

**Results:**

The base model and the 3D MPFDF achieved high fidelity in replicating the bone structures, accurately maintaining the morphological characteristics and dimensions such as length, width, and thickness, closely resembling natural bone. The radiographs of the 3D MPFDF displayed distinct radiopaque and radiolucent areas, enabling clear visualization of the various anatomical structures of the femur. However, in these radiographs, it was challenging to distinguish between the cortical and medullary regions due to the use of 99% internal padding in the printing process. Despite this limitation, the radiographs successfully demonstrated the representation of the Salter-Harris classification.

**Conclusions:**

This paper presents a pioneering project focused on technological advancement aimed at developing a method for the rapid and cost-effective production of three-printed models and radiographs of physeal fractures in dogs.

## Introduction

The femur is the most frequently fractured bone in dogs, with incidents ranging between 30 to 50%^1^^,^ 2. Such fractures can lead to lameness, functional impairment, or complete immobility of the affected limb^2^. Fractures can occur across all regions of the canine femur^3^, with the distal extremity accounting for 28% of cases^1^. Physeal fractures are categorized into grades I through VI according to the Salter-Harris classification system^4^. The diagnosis of these fractures typically involves a review of the trauma history, a physical examination, and, primarily, radiographs taken from at least two positions^5^^,^ 6.

Radiography is the preferred diagnostic method for assessing the anatomical location of a fracture, determining the appropriate treatment, and predicting the prognosis. Understanding these fractures is crucial for veterinarians, as it equips them to recognize and provide optimal clinical and surgical interventions^6^. Small animal practitioners are likely to encounter numerous long-bone fractures throughout their careers^1^. Veterinary students typically study these fractures through textbooks, medical images, and clinical cases^7^. Additionally, fracture models serve as valuable educational tools in veterinary medicine.

The adoption of three-dimensional (3D) printing in various aspects of veterinary medicine, including orthopedics^8^, diagnostic imaging^9^, and education^10^^–^ 12, has become increasingly prevalent. This technology offers high-quality teaching materials^12^^,^ 13 and can enhance understanding of real anatomy in details, providing an alternative to using live animals. This study aimed to create 3D models of physeal fractures in dog femurs (MPFDF) and evaluate the validity of these models through their radiographs.

## Methods

The study was carried out at the 3D Technologies Laboratory at the Universidade Federal do Acre. The methodology spanned three steps:

- Creation of 3D MPFDF;
- Radiographic study of 3D MPFDF;
- Anatomical and radiographic comparative analysis of 3D MPDF.

The study was registered and approved under process number 23107.007273/2017-49 by the Ethics Committee on the Use of Animals of the Universidade Federal do Acre.

### Creation of three-dimensional models of physeal fractures in dog femurs

**Figure 1:**
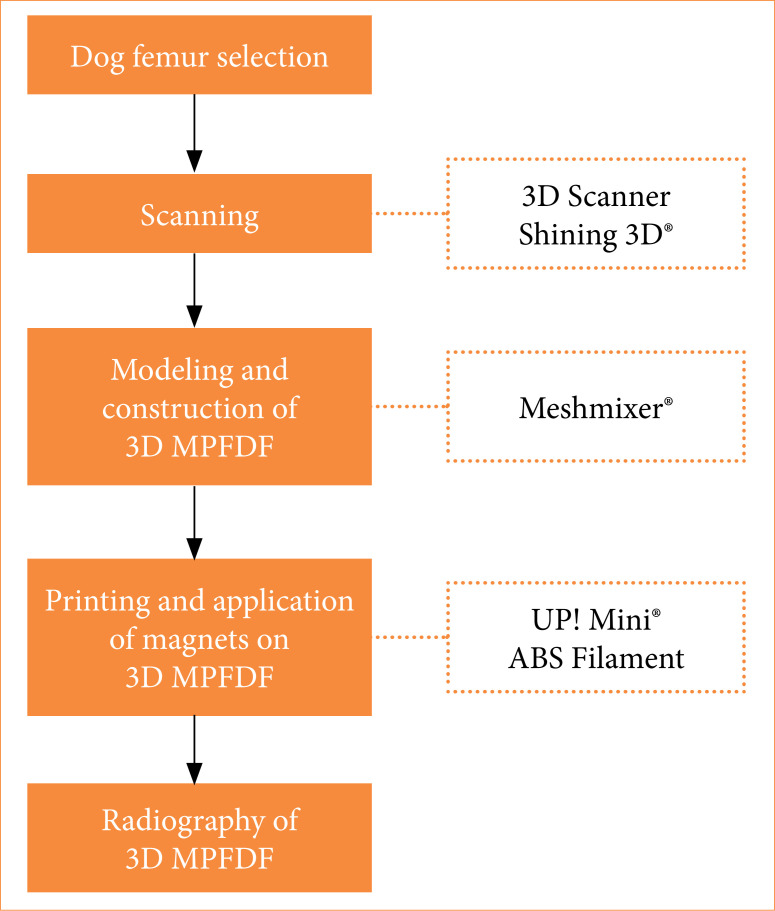
Flowchart of the three-dimensional canine physeal fracture model creation.

Cadaver femurs from healthy dogs that died of natural causes were macerated and prepared for scanning and subsequent 3D printing. The digital file obtained from scanning the original bone was used to create the base 3D model. This model was then employed to generate the 3D MPFDF, which illustrated the classification of physeal fractures (Fig. 1). The femur bone was scanned using a 3D scanner (Model EinScan-SP, Shining 3D, Zhejiang, China), and the scans were interpreted using the EinScan-SP Version 2.6.0.8 software, provided with the scanner. The scanner’s cameras captured the surface of the bone, acquiring images that were rendered into a mesh composed of thousands or even millions of triangles.

3D MPFDF: three-dimensional models of physeal fractures in dogs’ femur; ABS: acrylonitrile butadiene styrene. Source: Elaborated by the authors.

The captured images were saved in .stl format and stored in a database. They were uploaded to Autodesk Meshmixer, version 3.1 (Autodesk Inc., CA, United States of America), 3D creation and manipulation software, for the modeling and composition of the 3D MPFDF. This software facilitated the correction of the generated images, offering tools to remove uneven surfaces, smooth meshes, reduce noise, fill flaws, and create holes for magnet placement.

The modeling phase involved delineating the anatomical regions prone to fractures, ensuring that no information was lost during this process, and all femur structures were preserved. The fracture locations reproduced in the 3D MPFDF were based on those documented by Vulpe^4^ and Engel and Kneiss^14^ (Table 1).

**Table 1:** Features of the physeal fractures per the Salter-Harris classification.

Type	Features
I	Fracture through the physis.
II	Fracture across the physis that extends into a portion of the metaphysis.
III	Fracture involving the physis that extends into a portion of the epiphysis.
IV	Fracture across the physis, epiphysis, and metaphysis.
V	Compression injury of the physis.
VI	Periosteal bridging between the metaphysis and epiphysis.

Source: Adapted from Vulpe^4^ and Engel and Kneiss^14^.

The constituent parts of each 3D MPFDF were fabricated using the UP Mini 3D printer (Beijing Tiertime Technology Co., Beijing, China), which employs high-quality fused deposition modeling technology and uses acrylonitrile butadiene styrene (ABS)-grade thermoplastic material. The models were printed with a 99% internal fill and a layer thickness of 0.15 mm. Following the printing, manual finishing was applied to refine the models. Neodymium magnets, each 4 mm in diameter and 2 mm in height, were inserted along the fracture lines of each model segment, facilitating easy assembly and disassembly.

### Radiographic study of three-dimensional models of physeal fractures in dog femurs

After the construction phase was completed, the models were transported to the diagnostic imaging center for radiographic imaging. The 3D MPFDF were radiographed using an Emic Limex machine, set at a radiation intensity of 48 kV with an exposure time of 3 seconds. The models were positioned in two orientations recommended by Schachner and López^5^ for long-bone fractures: craniocaudal (CC) and lateromedial (LM). The resulting images were processed using Carestream Image Suite 4.0. For the radiographic examination, it was necessary to reprint the models with a 99% internal filling, but without the neodymium magnets. During this preparation, transparent double-sided adhesive tape was applied to the fracture surfaces of the models to enhance the visibility of the fractures while maintaining the integrity of the model during imaging.

### Anatomical and radiographic comparative analysis of three-dimensional models of physeal fractures in dog femurs

The models underwent a validation process through a comparative analysis with their corresponding radiographs. The primary objectives of this comparison were to confirm the presence of anatomical structures in the models and to assess whether various types of fractures were accurately depicted in the radiographic images. Additionally, this comparison served to identify any potential limitations encountered when radiographing 3D models, such as issues with image clarity or the visibility of specific fracture details.

## Results

**Figure 2:**
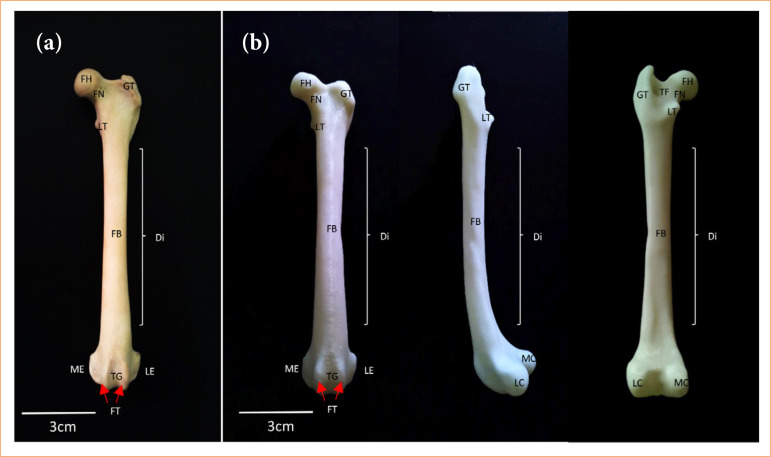
Femur of dog. **(a)** *In natura*: cranial view; **(b)** three-dimensional base model: cranial, lateral and caudal view, respectively.

FH: femoral head; FN: femoral neck; GT: great trochanter; LT: lesser trochanter; FB: femoral body; Di: diaphysis; ME: medial epicondyle; TG: trochlear groove; LE: lateral epicondyle; FT: femoral trochlea; MC: medial condyle; LC: lateral condyle; TF: trochanteric fossa. Source: Elaborated by the authors.

The 3D base model of the femur closely mirrored the conformation of the natural bone, accurately maintaining identical length and width and replicating key identifying structures. The anatomical accuracy of the model was demonstrated by the distinct visibility of several critical features, which include the femoral head, femoral neck, greater trochanter, lesser trochanter, trochanteric fossa, femoral body, trochlear groove, femoral trochlea, lateral epicondyle, medial epicondyle, lateral condyle, and medial condyle (Fig. 2).

Scanning the femur bone to create the 3D base model required only 5 minutes. The time for creation and printing, as well as the amount of material used and the overall costs of printing, varied depending on the complexity of each model and the inclusion of holes for magnet placement (Table 2). Among the models, the 3D MPFDF-Type V required the longest creation time. Conversely, the 3D MPFDF-Type IV and VI models demanded the most time for printing, reflecting the detailed intricacies involved in their design. This variation highlights the influence of model complexity on the resources and time required for production.

**Table 2:** Creation time, print time, quantity of material used, and costs of the three-dimensional models of physeal fractures in dog femurs.

3D MPFDF	Creation time (min)	Print time (h)	Material used (g)	Cost (US$)
Type I	45	4.6	22.1	0.67
Type II	30	4.5	21.8	0.66
Type III	35	5.6	27.3	0.82
Type IV	60	5.8	27.4	0.83
Type V	90	4.8	22.0	0.66
Type VI	20	5.8	28.3	0.85
**TOTAL**	**280**	**31.1**	**148.9**	**4.49**

Source: Elaborated by the authors.

The printing costs primarily stemmed from the amount of filament used, which accounted for 70% of the total expense. The remaining 30% was attributed to machine depreciation and electricity consumption. However, it is important to note that the costs associated with the equipment (3D printer and 3D scanner) were not included in this calculation.

**Figure 3:**
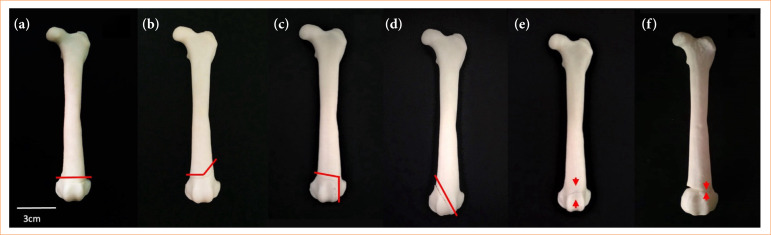
Three-dimensional anatomical models of the dog fractures physeal. **(a)** Type I, **(b)** type II, **(c)** type III, **(d)** type IV, **(e)** type V, **(f)** type VI. Red lines indicate fracture foci. Red flow indicates compression injury or bridging between the metaphysis and epiphysis.

Source: Elaborated by the authors.

Six different 3D MPFDF were printed, each representing distinct types of fracture (Fig. 3). These are as follows:

- The 3D MPFDF-Type I model featured a cut in the distal region perpendicular to the longitudinal axis of the model bone, dividing it into two sections: one comprising the proximal and middle thirds, and the other containing the distal third;
- The 3D MPFDF-Type II model included two cuts in the distal region. The first cut was made in the metaphysis, starting from the medial aspect to the intermedium region, followed by a diagonal cut toward the proximal region on the lateral aspect of the model bone. Two magnets were placed in each part of the model to facilitate assembly and disassembly;
- In the 3D MPFDF-Type III model, two cuts were also made in the distal region. The fracture path traversed the medial side of the physis and continued through the epiphysis into the joint. Four inserts were incorporated into each segment of the bone to allow for magnet insertion and enhanced stability;
- For the 3D MPFDF-Type IV model, one diagonal cut in the proximal region of the medial aspect in the direction until the distal intermedium region of the model bone was made. Three inserts were created in each portion for the insertion of the magnet with excellent stability;
- In the 3D MPFDF-Type V model, a groove representing the crushing or collapsing of the growth plate involving both the medial and lateral sides was created on the surface of the bone. This groove was located in the distal region and ran perpendicular to the longitudinal axis of the model bone. It was unnecessary to add magnets to this model, as the design did not require the bone to be divided into separate portions, allowing for a continuous, single-piece representation of the fracture type.
- In the 3D MPFDF-Type VI model, a cut was made on the medial side, which then transitioned to a groove on the lateral side in the distal region. Additionally, an increased volume was created on the lateral side to represent the development of a periosteal bridge connecting the metaphysis and epiphysis. Since this model did not require segmentation into separate parts, the inclusion of magnets was unnecessary, allowing for a seamless and integrated representation of this specific fracture type.

**Figure 4:**
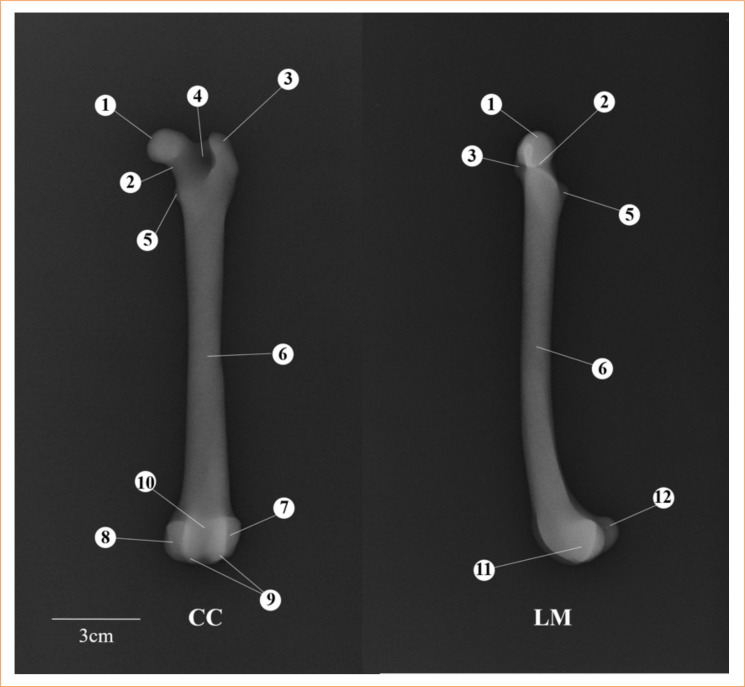
Radiographs of the three-dimensional base model. Craniocaudal (CC) and lateromedial (LM) views. (1) Femoral head, (2) femoral neck, (3) great trochanter, (4) trochanteric fossa, (5) lesser trochanter, (6) femoral body, (7) lateral epicondyle, (8) medial epicondyle, (9) femoral trochlea, (10) trochlear groove, (11) lateral condyle, (12) medial condyle.

Source: Elaborated by the authors.

The radiographic images of the 3D MPFDF displayed anatomical structures characteristic of the femur, including the femoral head, femoral neck, greater trochanter, lesser trochanter, trochanteric fossa, femoral body, lateral epicondyle, medial epicondyle, femoral trochlea, trochlear groove, lateral condyle, and medial condyle (Fig. 4). However, across all radiographs of the 3D MPFDF, it was not possible to distinguish between the cortical and medullary regions of the model bone, indicating a limitation in the imaging detail of these particular areas.

**Figure 5:**
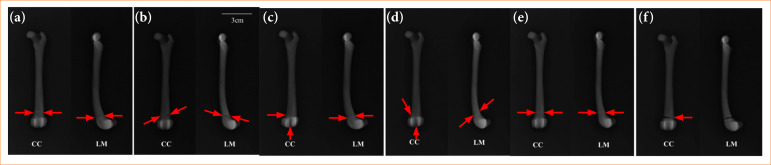
Radiographs of three-dimensional anatomical models of the dog fractures physeal representing the types I, II, III, IV, V, and VI according to Salter-Harris classification. Craniocaudal (CC) and lateromedial (LM) views. Red arrows indicate a fracture.

Source: Elaborated by the authors.

In the radiographs of the 3D MPFDF-Type I (CC and LM views), the fracture was depicted by a radiopaque line in the distal portion of the model, running perpendicular to the longitudinal axis (Fig. 5). This visualization effectively represented the fracture, demonstrating the model’s accuracy in mimicking the actual fracture dynamics in a canine femur.

For the 3D MPFDF-Type II, the radiography in the CC view displayed the fracture as an oblique radiopaque line running from the distal medial side toward the proximal lateral side of the model. In the LM view, the radiopaque line extended from the cranioproximal side to the caudodistal side of the model. These images effectively illustrate the directional nature of the fracture, showcasing the model’s ability to accurately represent different fracture orientations in radiographic evaluations.

In the X-ray of the 3D MPFDF-Type III (CC view), the fracture is depicted by two radiopaque lines. The first line is located in the distal portion, running perpendicular to the longitudinal axis of the model, extending from the medial to the intermediate portion. The second line starts perpendicular to the first one, extending along the distal end of the model. In the LM view, only the line in the distal portion perpendicular to the longitudinal axis is visible. In the radiography of 3D MPFDF-Type IV (CC view), the fracture is represented by a single oblique radiopaque line in the distal portion of the model, running from the proximal medial to the distal intermediate portion. In the LM view, the radiopaque line starts in the craniodistal view and ends in the caudoproximal view of the model.

The radiographs of 3D MPFDF-Type V (CC and LM views) show the alteration depicted by a radiolucent line in the distal portion, perpendicular to the longitudinal axis of the model. The 3D MPFDF-Type VI radiography (CC view) reveals the alteration represented by a radiopaque line initiating in the medial view, followed by an increase in radiolucent volume (representation of a bridge) in the side view. In the LM view, a radiopaque line is observed in the distal portion of the model, perpendicular to its longitudinal axis.

## Discussion

The knowledge of physeal fractures in dogs’ femur is relevant since this condition is common in veterinary medicine^2^. A retrospective study showed the frequency of Salter-Harris fractures as 39.9% for type I, 37.8% for type II, 3.1% for type III, and 19.1% for type IV^14^. However, there is a noted scarcity of educational materials on 3D models for teaching these fractures, underscoring a significant gap in educational resources^13^^,^ 15.

Current market offerings of didactic anatomical models lack biological variation and pathological authenticity, potentially leading to incorrect diagnoses and practices in real clinical scenarios^16^. Specifically, no anatomical MPFDF are available for sale. Utilizing 3D printing technology allows for a more accurate evaluation of femur deformities by providing palpable models that replicate the actual bone anatomy, presented as real-size, manipulable 3D structures^17^.

The scanning of the femur bone marks the initial stage in the production process of 3D MPFDF, preserving the primary anatomical references of the canine femur. This is similarly emphasized in other studies that described the importance of 3D scanning for developing models of jaw fractures and hip dysplasia in canines^12^^,^ 18. While our base model provided a good anatomical representation of the dog’s femur, it did not sufficiently reproduce some structures like the lateral and medial supracondylar tuberosities and the intercondyloid fossa. These observations align with Alcantara et al.^15^, who reported the loss of anatomical references in scanned and 3D printed models of dogs’ long bones in the pelvic limb. Another limitation was the non-visualization of the medullary cavity, a drawback inherent to the imaging method, as 3D scanners capture only the surface of the bone^19^^–^ 21.

Despite the limited visualization of some structures, these shortcomings do not impact the educational effectiveness of the 3D MPFDF. This is supported by Thomas et al.^10^, who, despite some loss of foramina and bone details, demonstrated that 3D models are viable for teaching anatomy. The creation time for the 3D MPFDF was slightly longer (280 min) compared to a similar project by Nunez et al.^18^, which had a total creation time of 240 min. The additional time in our study was required to create places for the insertion of magnets and to represent the fracture lines accurately.

The creation times for the 3D MPFDF models (types I, II, III, IV, and VI) were generally similar, except for type V, which took the longest due to the complexity of representing the collapse of the growth plate (Salter-Harris type V) and the development of a periosteal bridge between the metaphysis and epiphysis (Salter-Harris type VI). The differences in creation times reflect the intricacies involved in accurately representing various fracture types and pathological changes.

The total printing time for the 3D MPFDF was approximately 30 h, significantly longer than the 7 h required to print 3D models of the canine skull^22^. This difference in printing time is directly influenced by the complexity of the models and the settings used during printing, such as the internal filling of the model, layer thickness, temperature, extruder nozzle^23^, and the structure of the model’s support^13^.

Although the initial investment in scanning and printing equipment is considerable, the subsequent cost for model production is relatively low^24^. In this study, 148.9 g of filament was used, and the total cost of 3D MPFDF did not exceed US$5, which compares favorably to the study by Nunez et al.^18^, in which 653.55 g of thermoplastic filament (ABS) was used for printing a set of 3D models of canine hip dysplasia at a reported cost of US$20.25. Despite the differences in the parts printed, the type of material, amount used, and cost of printing were similar, and it is believed that these costs could be reduced further by using the highest resolution available on our 3D printer.

The inclusion of neodymium magnets in the fracture lines of each segment of the 3D MPFDF (Salter-Harris types I, II, III, and IV) facilitated the assembly and disassembly of the parts. This modularity is crucial as it allows for easier manipulation of the models and a more detailed demonstration of the fractures. Our findings align with those of Preece et al.^9^, who suggested that physical models may offer significant advantages over other learning resources in enhancing visuospatial understanding and comprehension of complex 3D anatomical structures.

According to data from Engel and Neiss^14^, the prevalence of fractures in the distal region is significant (79.5 vs. 20.5%). Consequently, we chose to reproduce the representations of the Salter-Harris classification specifically in the distal region of the model. Researchers have demonstrated that the use of anatomical models in conjunction with other teaching methods significantly enhances student skill development^25^^,^ 26. Thus, we also utilized radiographs as a tool to objectively assess the quality of the 3D MPFDF before their use in educational settings.

The use of two radiographic positions (CC and LM) for each 3D MPFDF proved sufficient. This combination of positions allowed for the visualization of fracture lines (Salter-Harris types I, II, III, and IV), the collapse of the growth plate (Salter-Harris type V), and the development of a periosteal bridge between the metaphysis and epiphysis (Salter-Harris type VI). These positions are commonly used to diagnose physeal fractures in dogs^4^.

The radiopacity and radiolucency of the 3D MPFDF do not correspond to the bone densities typically seen in the radiography of natural canine femurs. In practice, the density of the models was directly influenced by the type of material (thermoplastic) and the maximum internal fill (99%) used in their manufacturing. Using an internal fill of less than 99% would create visible internal grids on the radiographs, which are artifacts that obscure the visualization of the fracture lines in the models^12^.

The creation of radiographs from the 3D MPFDF without neodymium magnets was necessary due to initial tests indicating that magnets interfered with the visualization of the fracture lines. Similar issues were reported by Lima et al.^12^, who observed artifacts when attempting to X-ray 3D models of canine jaw fractures that included magnets. The radiographic images produced from the 3D MPFDF effectively represented the classification of physeal fractures in the canine femur. However, these models were not suitable for surgical training purposes, as they could not replicate the different bone densities, such as compact and cancellous bone, which are crucial for understanding how to stabilize fractures with plates and screws using knowledge of bone density and the dissipation of forces along the trabeculae of spongy bone^6^^,^ 27.

The creation of these radiographs provided a valuable resource for demonstrating radiographic aspects not typically studied. Furthermore, the combination of 3D MPFDF and their corresponding radiographs offers a unique educational tool. This setup allows future veterinarians to manipulate the physical model while simultaneously observing its radiographic image, enhancing their understanding of the disease in its various manifestations. This interaction between palpation and visual analysis has the potential to greatly enhance students’ comprehension of the studied content^9^^,^ 28.

## Conclusion

This study presents a pioneering and technologically advanced project focused on developing a method for the rapid and cost-effective production of 3D printed models and radiographs of physeal fractures in dogs. The 3D MPFDF and their respective radiographs successfully replicated the anatomical structures and fracture lines typical of the condition. These models hold significant potential primarily for use in educational settings, particularly in the disciplines of anatomy, surgery, and diagnostic imaging. This initiative not only enhances the learning experience by providing tangible, manipulable models, but also contributes to a deeper understanding of complex veterinary conditions.

## Data Availability

All data sets were generated or analyzed in the current study.
